# Jejunal Intussusception: A Rare Manifestation of a Primary Thyroid Non-Hodgkin Lymphoma

**DOI:** 10.7759/cureus.2717

**Published:** 2018-05-31

**Authors:** Abu Baker Sheikh, Usman Tariq, Marvi M Bukhari, Sana Shah, Rao M Afzal, Abdul Ahad E Sheikh, Nimra Nadeem

**Affiliations:** 1 Internal Medicine, University of New Mexico, Albuquerque, USA; 2 Research Assistant, Yale University School of Medicine, New Haven, USA; 3 Internal Medicine, Shifa College Of Medicine, Islamabad, PAK; 4 Student, Aga Khan University Hospital, Karachi; 5 Student, Shifa College Of Medicine, Islamabad, PAK

**Keywords:** non-hodgkin lymphoma, intussusception, airway compression, thyroid cancer

## Abstract

Primary thyroid lymphoma (PTL) is an uncommon malignancy of the thyroid gland, with most lymphomas of the thyroid being almost exclusively of the non-Hodgkin's B cell variety. PTL requires a prompt diagnosis because of its ability to cause progressive compression symptoms, and its unusual presentation can make the diagnosis very challenging. Herein, we present a case of PTL in a young woman with an uncommon initial presentation and discuss the complications she faced during the surgery, as well as postoperatively, due to the compression of the trachea by the thyroid mass.

## Introduction

Primary thyroid lymphoma (PTL) is an exceptionally rare malignancy. Nevertheless, it is imperative to be well versed with its clinical presentation, which inevitably aids in making a prompt diagnosis and ensuring appropriate treatment. Thyroid lymphoma is classified under the banner of non-Hodgkin lymphoma (NHL) and it represents approximately 1.2% to 1.7% of all NHLs. It constitutes < 5% of all thyroid malignancies and < 2% of extra-nodal lymphomas [1]. Females have a three to a four-fold higher predisposition to this ailment, which is frequently seen in those afflicted with Hashimoto’s thyroiditis. It ordinarily presents in their sixth or seventh decade of life. It also permeates the male population and adolescents, albeit less frequently [2].

## Case presentation

A 19-year-old female presented to our hospital with complaints of vomiting for one week, along with generalized abdominal pain and weight loss for the last three months. Initial assessment found the patient to be alert and well-oriented, albeit pale, emaciated, and considerably uncomfortable due to the pain. Her heart rate was 103 per minute with a blood pressure of 100/60 mm of Hg, a respiratory rate of 16 per minute, and a temperature of 98.4°F. She had conjunctival pallor. An abdominal exam revealed that she had a distended abdomen with generalized tenderness and a palpable mass in the epigastrium. Her hernial orifices were intact but there were no discernable bowel sounds on auscultation of the abdomen. A digital rectal exam revealed an empty rectal vault.

Laboratory investigations done in the emergency room revealed a low hemoglobin count of 7.2 g/dL, a platelet count of 650,000/µL, and a total leukocyte count of 11,400/µL. Her creatinine was 0.60 mg/dL with a blood urea level of 38 mg/dL. Her potassium level was 4.0 mEq/L and the international normalized ratio (INR) was 1.0. Owing to the patient's abdominal pain, she underwent an abdominal ultrasound scan, which revealed a jejunal intussusception with dilated loops of bowel, while a computed tomography (CT) scan of the abdomen showed a donut intussusception. She also underwent a CT scan of the chest that showed a mediastinal mass with a resultant compression of the trachea.

**Figure 1:**
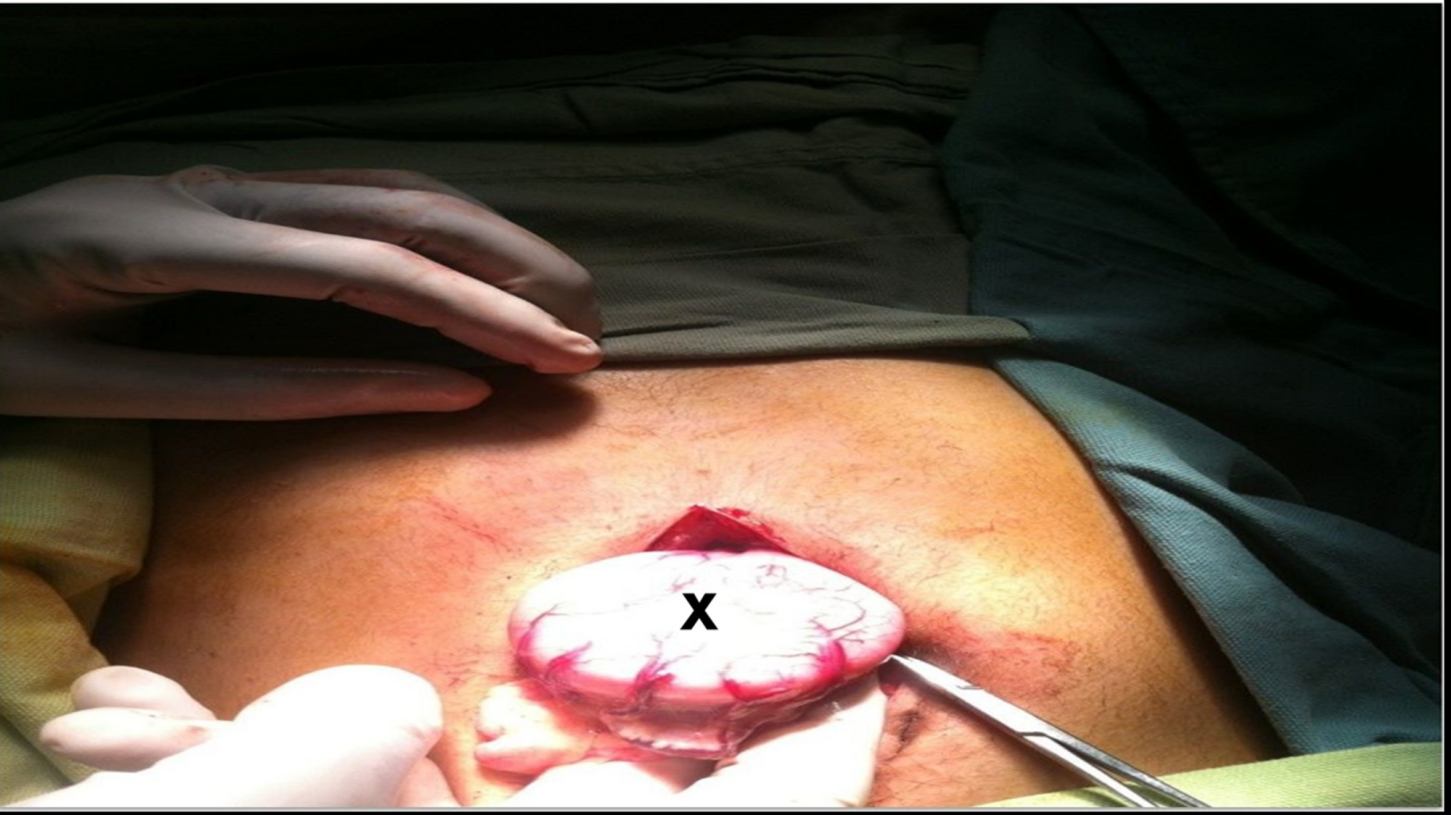
Dilated small bowel loop (marked by letter X)

**Figure 2:**
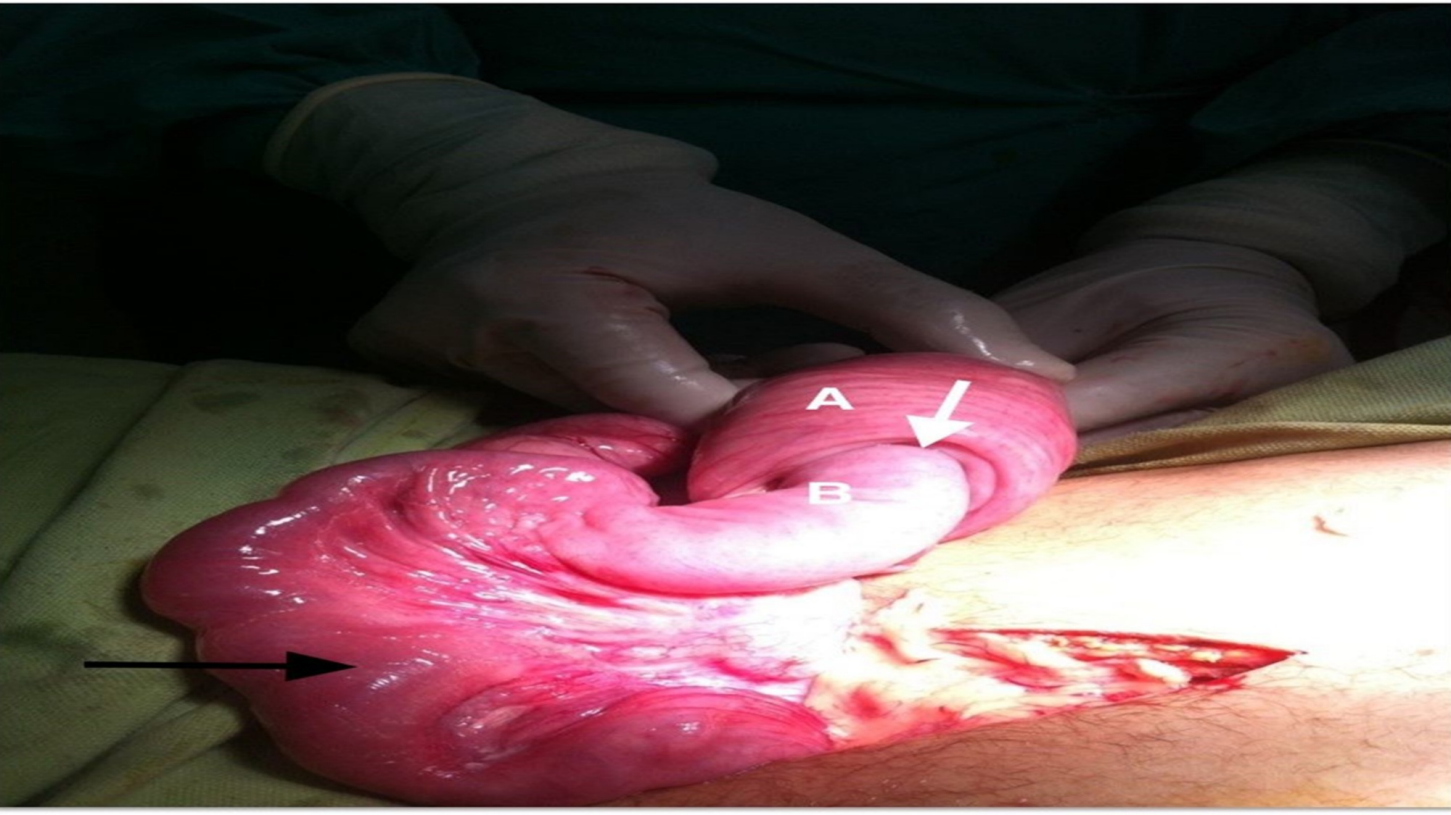
Small bowel of the patient during surgery Small bowel during surgery showing telescoping (white arrow) of the proximal segment (marked by B) into the distal segment (marked by A). There is dilation and edema of the proximal small bowel (black arrow)

**Figure 3:**
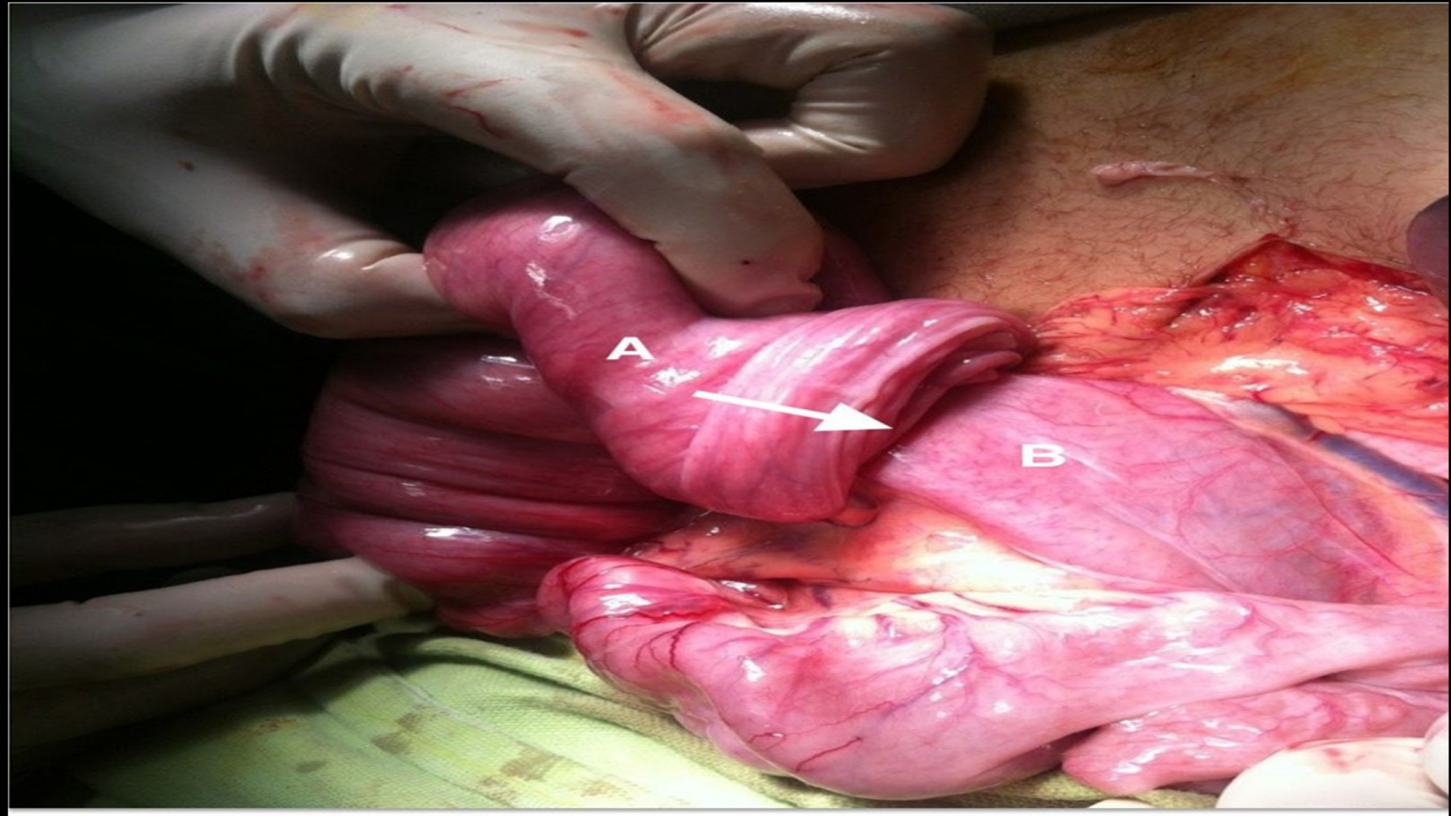
Small bowel of the patient during surgery Small bowel during surgery showing telescoping (white arrow) of the proximal segment (marked by B) into the distal segment (marked by A)

Due to this clinical presentation, she was admitted for a surgical intervention. A difficult endotracheal intubation, owing to the mass causing tracheal compression, was eventually followed by an emergent laparotomy. A proximal jejunal intussusception with dilated loops of bowel was observed during the procedure, which culminated in an ileotransverse bypass (Figures 1-3).

During the surgery, the patient experienced recurrent episodes of non-ventilation due to tracheal pressure, which improved with repositioning of the endotracheal tube. Following the procedure, she was admitted to the intensive care unit. Her issues with ventilation continued to linger in the postoperative period but her oxygen saturation improved yet again with a repositioning of the endotracheal tube.

Unfortunately, the patient died the following morning due to an episode of sudden apnea. A biopsy of the mediastinal mass revealed an aggressive non-Hodgkin lymphoma of the thyroid gland.

## Discussion

PTL regularly presents with an enlarging neck mass that precipitates symptoms, such as dyspnea, dysphagia, and hoarseness, owing to the mass effect of the tumor in the vicinity of adjacent soft tissues. Other patients may present with B-cell symptoms, such as fever (> 38° C) and weight loss (> 5% from baseline), which, if present, may add to the poor prognosis of a patient with NHL [3-4].

NHL can arise as a primary disease of the lymph nodes or display as a malignancy at extranodal sites. More than half the patients have some degree of extranodal involvement at the time of their initial diagnosis. Based on these attributes, the malignancy may raid any organ system. NHL can affect the gastrointestinal tract, a phenomenon observed in 10% - 30% of all patients with the disease [5]. However, bowel intussusception in the adult demographic is uncommon and accounts for only 5% of the total tally of intussusceptions observed. Furthermore, most cases are mild with only 1% - 5% of the patients eventually deteriorating to subsequent intestinal impedance [6]. We present the case of a metastatic thyroid lymphoma that hastened the development of a proximal jejunal intussusception.

Akbulut reviewed 36 cases of adult intussusception due to lymphoma. Twenty-nine patients were male and seven were female, which deviates from the common epidemiology of NHL. Twenty-four patients had ileocolic intussusception and 10 had enteric, while two had colocolic intussusception. In terms of the diagnosis, 34 patients were diagnosed with various types of NHL, and two patients were diagnosed with Hodgkin lymphoma [7]. In our literature review, we found a paucity in the number of publications that describe intestinal intussusception as an initial clinical presentation of an NHL, which underscores the rarity of this clinical picture [8].

Our patient was diagnosed on the basis of her computed tomography (CT) scan, which is the gold standard for identifying intussusceptions. A subsequent laparotomy with bowel resection is the endorsed course of action in cases where malignancy is the underlying culprit, such as in our patient [9].

The unfortunate and unforeseen demise of our patient was ascribed to respiratory insufficiency in the postoperative period. This was secondary to a difficult airway owing to the tumor mass, which ultimately led to an apneic collapse. Through our discussion, we would like to reiterate the notion of an adequate assessment of the airway prior to and following an urgent surgery. This includes obtaining an adequate history, if possible, to assess the severity of airway compromise, followed by the application of a topical anesthetic to the upper airway while the patient is alert, and its subsequent assessment via a rigid laryngoscope. Also, while the patient is awake, make use of techniques, such as fiberoptic-guided or retrograde tracheal intubations, to establish an airway. Failure to do so could warrant a tracheostomy with local anesthesia to establish adequate airflow during an emergent surgical procedure. Performing an extubation following a prolonged surgery, as in our case, could be complicated because the mass effect of the tumor and a superimposed soft tissue edema of the trachea could hamper airflow. It is, therefore, recommend to only extubate after the edema has resolved and, even then so, over a jet ventilation stylet [10].

## Conclusions

Primary thyroid lymphoma can present with a diverse assortment of clinical presentations, which signifies the importance of having a high measure of clinical suspicion that facilitates a swift diagnosis and its subsequent treatment. An emergent surgery is an option in the management of a severe intussusception. The unfortunate clinical outcome in our setting underscores the importance of an appropriate management of the airways in the background of head and neck malignancies.
